# The Association of the Colleges of Dentistry

**Published:** 1868-05

**Authors:** 


### EDITORIAL DEPARTMENT.

*The Association of the Colleges of Dentistry.*—At a meeting of the Association of the Colleges of Dentistry held in New York on the 2nd of April last, Section IV. of the By-Laws which read, "That eight years of Dental Practice, including a regular pupilage, will be regarded as equivalent to one course of lectures," was so amended as to read: "*That eight years of Dental Practice, or an acceptable examination upon Anatomy, Physiology, Inorganic Chemistry and Practical Dentistry, including a regular course of pupilage will be regarded as equivalent to one course of lectures.*

We expected to have received from the Secretary of the Association a report of the proceedings of the late meeting, but as it has not come to hand at the time of our going to press we shall not be able to present it to our readers in the present number.

